# Plant-mediated interspecific horizontal transmission of an intracellular symbiont in insects

**DOI:** 10.1038/srep15811

**Published:** 2015-11-13

**Authors:** Elena Gonella, Massimo Pajoro, Massimo Marzorati, Elena Crotti, Mauro Mandrioli, Marianna Pontini, Daniela Bulgari, Ilaria Negri, Luciano Sacchi, Bessem Chouaia, Daniele Daffonchio, Alberto Alma

**Affiliations:** 1 Dipartimento di Scienze Agrarie, Forestali e Alimentari (DISAFA), Università degli Studi di Torino, 10095 Grugliasco; 2 Dipartimento di Scienze per gli Alimenti, la Nutrizione, l′Ambiente (DeFENS), Università degli Studi di Milano, 20133 Milan; 3 Dipartimento di Scienze della Vita, Università di Modena e Reggio Emilia, 41100 Modena; 4 Dipartimento di Scienze Agrarie e Ambientali—Produzione, Territorio, Agroenergia, Università degli Studi di Milano, 20133 Milan; 5 Dipartimento di Biologia e Biotecnologie “L. Spallanzani”, Università degli Studi di Pavia, 27100 Pavia; 6 King Abdullah University of Science and Technology, BESE Division, Thuwal, 23955-6900, Kingdom of Saudi Arabia

## Abstract

Intracellular reproductive manipulators, such as *Candidatus* Cardinium and *Wolbachia* are vertically transmitted to progeny but rarely show co-speciation with the host. In sap-feeding insects, plant tissues have been proposed as alternative horizontal routes of interspecific transmission, but experimental evidence is limited. Here we report results from experiments that show that *Cardinium* is horizontally transmitted between different phloem sap-feeding insect species through plants. Quantitative PCR and *in situ* hybridization experiments indicated that the leafhopper *Scaphoideus titanus* releases *Cardinium* from its salivary glands during feeding on both artificial media and grapevine leaves. Successional time-course feeding experiments with *S. titanus* initially fed sugar solutions or small areas of grapevine leaves followed by feeding by the phytoplasma vector *Macrosteles quadripunctulatus* or the grapevine feeder *Empoasca vitis* revealed that the symbionts were transmitted to both species. Explaining interspecific horizontal transmission through plants improves our understanding of how symbionts spread, their lifestyle and the symbiont-host intermixed evolutionary pattern.

The intracellular bacterium “*Ca.* Cardinium hertigii”^1^ (hereafter *Cardinium*) is an intriguing arthropod symbiont capable of reproductive manipulation^2^,^3^,^4^,^5^ or of determining effects ranging from stimulation of the host’s immune response^6^, to fecundity benefits^5^,^7^, or even to reduction of the host’s fitness^8^.

*Cardinium* is a common symbiont of insects^9^,^10^, mites^11^, spiders^12^,^13^, and opilionids^14^. *Cardinium* is maintained in a given host population via maternal transmission to progeny through the egg cytoplasm. However, vertical transmission is not sufficient to explain the observed co-speciation patterns with insect hosts. Moreover, various *Cardinium* lineages are shared with different and distantly related host species^9^,^15^. Such a patchy evolutionary pattern indirectly suggests that *Cardinium* may also exchange hosts via horizontal transmission patterns^16^.

Horizontal transmission has been observed in several host models. Aphid symbionts may be transferred orally when individuals share the same food source^17^,^18^,^19^. Environmentally acquired symbiotic *Burkholderia* are transferred among individuals of the broad-headed insect *Riptortus clavatus*^20^. The actinobacterial symbiont *Coriobacterium glomerans* of the red firebug *Pyrrhocoris apterus* and the symbionts of the firebrat *Thermobia domestica* are acquired horizontally by juveniles through symbiont-containing eggshells, faeces, or adult bugs^21^,^22^. *Rickettsia* symbionts of the whitefly *Bemisia tabaci* can be acquired by its parasitoids^23^ or by other whiteflies feeding on the same plant^24^.

Phylogenetic analyses of *Wolbachia* a reproductive manipulator, and its associated hosts showed horizontal transmission between prey and predator mites^25^ and between hosts and parasitoids^26^, as well as among insects sharing the same food source or parasites^27^,^28^. Direct demonstrations of intra- or inter-specific horizontal transmission of *Wolbachia* from the host to a parasitoid^29^, or in parasitoids sharing the same host eggs^30^,^31^, have been reported in few host models. Horizontal transmission has also been directly or indirectly observed in *Arsenophonus nasoniae*, which is transferred among parasitic wasps sharing the same hosts^32^. On the other hand, in the case of *Cardinium*, there has only been speculation on possible horizontal transmission^9^,^10^,^13^,^16^,^33^,^34^,^35^.

Here we demonstrate that an inter-specific horizontal transmission of *Cardinium* can take place in leafhoppers and that such a transmission occurs through the plant tissue pierced by the insect host. By using the cicadellid *Scaphoideus titanus*, a *Cardinium*-holder strictly feeding on grapevine leaves, as a model, we show that *Cardinium* is released from the insect’s salivary glands to the plant tissues and then horizontally acquired by other grapevine feeder and non-grapevine feeder insects.

## Results

### *Cardinium* release in artificial feeding media and plant tissue

The first set of experiments was conducted to assess the capability of *S. titanus* to release *Cardinium* during feeding on an artificial substrate (a feeding medium consisting of a sterile sugar solution) or on grapevine leaves. *S. titanus* individuals used in the inoculation experiments without antibiotic treatment were found to be highly colonized by *Cardinium*. The infection rate and the concentration of *Cardinium* cells were very similar in insects fed on the artificial media and the grapevine leaves (Table 1). In rifampicin-treated insects fed on both substrates, *Cardinium* was found as well, and the average infection rate and density of symbiont cells per leafhopper were not significantly lower than those in untreated specimens. However, according to the *Cardinium*-to-bacteria ratio (CBR), the percentage of *Cardinium* in the whole leafhopper bacterial community after the rifampicin treatment was drastically decreased in both experimental settings (Table 1).

*Cardinium* was consistently found to colonize *S. titanus* individuals, which were found to be able to inoculate symbiont cells in either feeding substrate and in either the absence or the presence of antibiotic treatment. However, both inoculation/infection rates and titres decreased in the insects treated with rifampicin compared with those that were untreated regardless of the feeding substrate. These values consistently declined after antibiotic treatment in the sugar diet and the grapevine diet, and a statistical significance was detected between samples with or without rifampicin. The CBR calculated for the diet samples followed a similar decrease (Table 1).

While grapevine leaves collected from *S. titanus*-free plants were always negative for the bacterium, 34.3% of grapevine leaves collected from plants in the field infested by the leafhopper were *Cardinium*-positive (Table 1). Even so, the average infection rate and titre were not significantly higher than the average infection rate and titre of grapevine leaves exposed to antibiotic-treated *S. titanus*.

**Figure 1:**
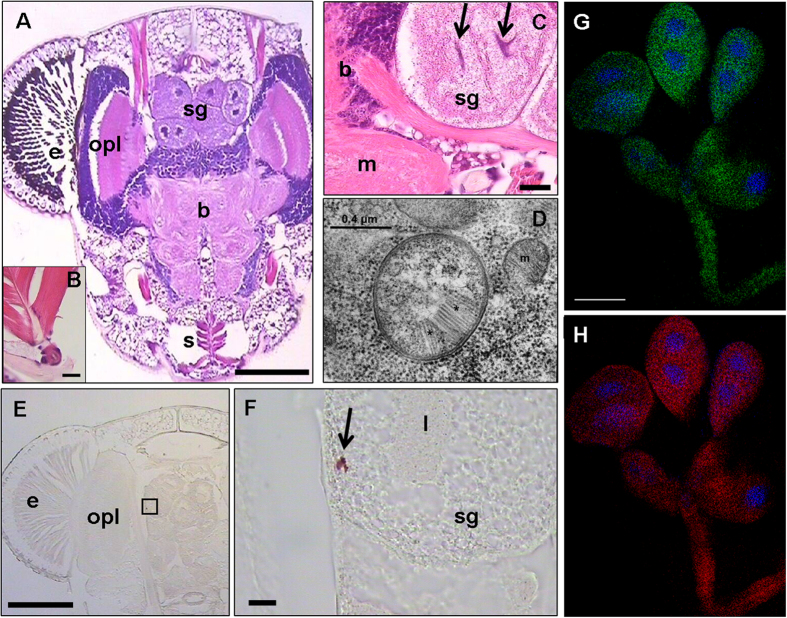
Morphology of the *S. titanus* head (including salivary glands) and occurrence of *Cardinium* in the salivary glands. (**A**) Section of the head of *S. titanus*, stained with hematoxylin/eosin. b = brain; e = eye; sg = salivary glands; opl = optical lobe; s = stylet. Bar = 0.25 mm. (**B**) Stylet, particularly showing cibarial pump muscles. (**C**) Salivary glands, particularly showing the lumen (arrows). b = brain; m = medulla; sg = salivary gland. Bar = 100 μm. (**D**) Micrograph of *Cardinium* showing the microtubular-like complex with numerous tubules (asterisks) and the Gram-negative architecture of the bacterium cell wall. m = mitochondrion. (**E,F**) ISH of the head (E, bar = 0.25 mm), showing positive staining (red) for *Cardinium* probes (arrow) in the salivary gland (F, particular of the framed part in E. Bar = 50 μm). e = eye; l = salivary gland lumen; opl = optical lobe; sg = salivary gland. (**G,H**) Confocal laser scanning microscopy images of salivary glands after hybridization with Cy5-labelled *Cardinium*-specific probes (G, green) and Texas Red-labelled eubacterial probes (H, red) probes. Blue spots indicate DAPI-stained gland nuclei. Bar = 50 μm.

**Figure 2:**
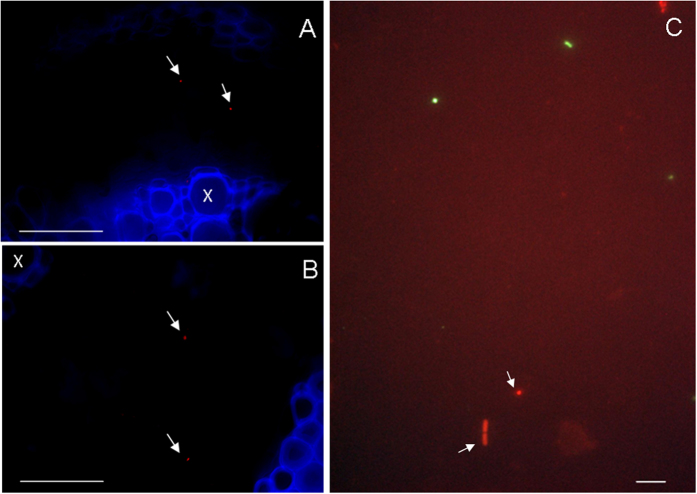
FISH experiments on feeding substrates after exposure to *S. titanus*. (**A,B**) Video-confocal micrographs of a grapevine leaf midrib after it was used by the leafhopper for feeding (**A**) and a field vine leaf portion (**B**) after hybridization with the Cy5-labelled *Cardinium* probes. Arrows indicate *Cardinium* cells within the phloem tissues (x = xylem). (**C**) FISH of a sugar diet exposed to *S. titanus*. Signals corresponding to both the FITC-labelled eubacterial probe (green) and the Texas Red-labelled *Cardinium*-specific probe (red) are visible. Bars = 50 μm (**A**) and (**B**); 2.5 μm (**C**).

We tested some of the salivary glands of *S. titanus* and the artificial feeding media and grape leaves using *in situ* hybridization (ISH) and fluorescence *in situ* hybridization (FISH). Hybridization signals related to the *Cardinium* probes were detected (Figs 1E–H, 2). TEM observations of the salivary glands of *S. titanus* confirmed the presence of bacterial cells with microtubular-like complexes typical of *Cardinium*^36^ (Fig. 1D). Moreover, FISH experiments on grapevine leaves collected in *S. titanus*-infested vineyards in the field also indicated the presence of *Cardinium* bacterial cells (Fig. 2B). On the other hand, when antibiotic-treated leafhoppers and their respective feeding substrates were tested by FISH, no hybridization signal was detected for *Cardinium.* Negative controls exhibited a lack of hybridization as well (Fig. S3).

### *Cardinium* transmission experiments

**Figure 3:**
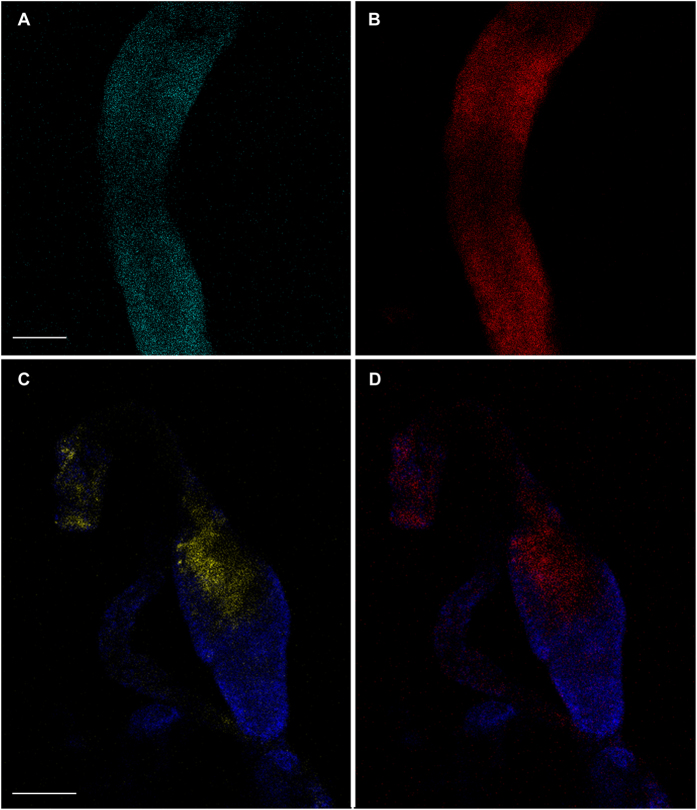
FISH experiments on midguts of leafhoppers sharing food substrates with *S. titanus*. (**A,B**) Midgut portion of *M. quadripunctulatus* after a three-day co-feeding on the artificial diet. The cyan signal corresponds to hybridization with the *Cardinium*-specific probes (**A**), while the red signal corresponds to the eubacterial probe (**B,C,D**) Midgut segment of *E. vitis* after sharing a grapevine leaf with *S. titanus* for 3 days. The hybridization with the *Cardinium*-specific probe is marked in yellow (**C**), whereas the eubacterial probe signal is stained in red (**D**). Intestine tissues are coloured with DAPI (blue). Bars = 75 μm.

Following the demonstration of the capability of *S. titanus* to inoculate *Cardinium* cells in its feeding medium, we tested if other insects could then acquire *Cardinium* from the feeding medium. We utilized the leafhoppers *Macrosteles quadripunctulatus* and *Empoasca vitis* as receiver hosts. Prior to the experiment, we assessed the *Cardinium* infection status of the recipient species. We did not detect the bacterium neither in *M. quadripunctulatus* nor in *E. vitis* individuals that had never been exposed to *S. titanus*’s feeding media. Transmission experiments *via* artificial feeding media (*M. quadripunctulatus*) or grapevine leaves (*E. vitis*) were then conducted. *Cardinium* was initially detected by qPCR in all *S. titanus* donor individuals. Moreover, in both cases, a transfer of *Cardinium* cells to *M. quadripunctulatus* and *E. vitis* was detected either by qPCR or by FISH experiments (Table 2; Fig. 3).

In the *M. quadripunctulatus* experiments, the *Cardinium* infection rate in recipients was initially about 50% (after one and three days of co-feeding) and then increased after one week. However, the average titres of *Cardinium* in positive individuals were modest with respect to donors in all experiments (with a peak after three days of co-feeding). The CBR in the recipient *M. quadripunctulatus* was low as well, following a similar trend of the titres (Table 2).

In the *E. vitis* experiments, both infection rates and concentrations were quite variable, with a peak after one day of co-feeding, a decrease after three days and a new increment after seven days. Interestingly, although the *Cardinium* concentration decreased in the seven-day trial, the CBR was higher for the longer co-feeding time; furthermore, *Cardinium* remained above 1% of total bacteria and always represented a higher percentage of the microbial community in *E. vitis* compared with *M. quadripunctulatus* (Table 2).

The transmission was confirmed by FISH and sequencing. Indeed, a massive hybridization signal correlated with *Cardinium* was detected in the midguts of *M. quadripunctulatus* and *E. vitis* after three days of co-feeding (Fig. 3A,C), whereas the negative controls were free of fluorescence signals related to probe hybridization (Fig. S4). Moreover, 835 bp amplicons were sequenced after the *Cardinium*-specific PCR was performed on both *M. quadripunctulatus* and *E. vitis* used in the co-feeding experiments. The sequences were identical to the deposited sequences of the *Cardinium* symbiont of *S. titanus* (AM042540), clustering in the A-group^1^ (Fig. S5).

## Discussion

This study sought to verify if *S. titanus* is able to inoculate *Cardinium* into the surrounding feeding substrate during its feeding activity and then if *Cardinium* in the feeding substrate can be taken up by other insects. By means of qPCR and FISH results, we firstly demonstrated that *S. titanus* injects the symbiont into its feeding substrates, both under controlled conditions (artificial diet) and in a semi-natural environment (grapevine leaves) (Table 1, Fig. 2). High concentration values of *Cardinium* were observed in the leafhoppers and in their artificial diet, in agreement with previous data^16^. Additionally, the detection of *Cardinium* in grapevine leaves, used for feeding by insects infected by the symbiont, suggests that *Cardinium* can spend part of its life cycle in grapevine leaf tissues, exploiting the plant as a means to be transmitted among *S. titanus* populations, as previously observed in other plant sap-feeders^17^,^18^,^24^. The observation of the symbiont in grapevine plants in the field where *S. titanus* is naturally present suggests that the leafhopper regularly releases *Cardinium* into the plant tissues, although infection rates and symbiont densities observed in the field were lower than those detected in grapevine leaves experimentally exposed to the leafhopper. This finding opens questions on the nature of the interaction between *Cardinium* and grapevine leaves, as well as the occurrence of the symbiont in other plants hosting sap-feeding insects, in light of the wide diffusion of this bacterial symbiont among arthropods^9^,^37^.

However, when *S. titanus* individuals were treated with rifampicin, we were not able to obtain completely *Cardinium*-free specimens. Moreover, these antibiotic-treated insects were still able to inoculate *Cardinium* into their feeding substrates (Table 1). The use of antibiotics was demonstrated to inhibit the occurrence of many symbionts completely^38^,^39^,^40^ even after a few days of administration^41^,^42^. On the contrary, symbiotic bacteria in some cases could still be found in their hosts after antibiotic treatment^42^,^43^. A reduction of the *Cardinium* titre by about one order of magnitude after rifampicin treatment was not as high as previously reported by Pajoro *et al.*^16^, while it was in the same range of the results obtained by Boucias and colleagues^43^ for *Burkholderia* in the heteropteran *Blissus insularis* Barber. Due to the impossibility of obtaining *Cardinium*-cured *S. titanus*, we employed different *Cardinium*-free leafhopper species as recipients to demonstrate the symbiont acquisition step. Both *M. quadripunctulatus* and *E. vitis* were able to acquire *Cardinium* that then persisted in the two recipient species (Table 2, Fig. 3).

*M. quadripunctultus*, which shared the artificial sugar diet with *S. titanus*, was stably colonized by *Cardinium* after feeding. Indeed, the symbiont infection rates observed during these transmission experiments increased at the longest time of exposure, indicating that longer co-feeding periods result in greater acquisition by the recipient. On the other hand, the *Cardinium* concentration in *M. quadripunctulatus* was always low and never reached values comparable with those detected in *S. titanus*, indicating that longer periods may be necessary to achieve stable colonization. It would be of great interest to elucidate whether *Cardinium* is able to persist in the new host and finally to be maternally transmitted. Moreover, because *M. quadripunctulatus* is known to be a very efficient phytoplasma vector^44^,^45^, this species may be particularly suitable for bacterial invasion of the salivary glands, leading to high transmission competence. The observation of the acquisition of *Cardinium* by *M. quadripunctulatus* raises a question about the capability of newly colonized insect individuals to transmit the acquired symbiont subsequently in the feeding medium and possibly to other insects.

Varying results were obtained both in terms of infection rates and density in the *E. vitis* experiments. After one day of co-feeding, a high percentage of the recipient leafhoppers exhibited considerable amounts of *Cardinium* uptake. However, after three days, the infection rates and concentrations declined and then increased again after seven days (Table 2). This variability suggests that the success of *E. vitis* colonization by *Cardinium* may be due to chance more than to a solid capability of the symbiont to invade the tissues of this insect. Hence, even though we demonstrated that at least in some cases the ingestion of *Cardinium* can be followed by successful colonization, the symbiont is likely to be infrequently established in *E. vitis*. Nonetheless, the symbiont concentration in *Cardinium*-positive *E. vitis* was always higher than that observed in *M. quadripunctulatus*, and it even reached values as high as those detected for *S. titanus* (Table 2). This evidence indicates that the tissues of grapevine leaves are suitable media for the acquisition of high titres of this bacterium, possibly because *Cardinium* is concentrated in the phloem of leaves and is more likely to be ingested than *Cardinium* from the artificial diet in which the bacteria are dispersed throughout the sugar solution. Since we never found *Cardinium*-positive field-collected *E. vitis*, the transmission from *S. titanus* to this leafhopper *via* grapevine leaves is probably not persistent, and the bacterium may not find optimal conditions in the new host to be then vertically transferred to its progeny. However, given that *Cardinium* was detected in grapevine leaves in the field, it is apparent that this plant may be a continuous reservoir of the symbiont, at least for *S. titanus*, and acquisition of this bacterium by other phloem-feeding insects residing on grapevine leaves cannot be excluded.

The overall results provide direct demonstration of the horizontal transmission of *Cardinium* in phloem-feeding leafhoppers *via* co-feeding, previously widely hypothesized^9^,^34^,^35^ but never demonstrated for this symbiont. The data presented demonstrate and support previous molecular evidence that horizontal transfer of symbionts does actually occur and may contribute to symbiont spread in natural insect lineages^9^,^28^,^35^. Even though maternal transmission is the main diffusion system for reproductive manipulators such as *Cardinium*, we showed that horizontal transmission through plants provides an alternative route of spread of these symbionts. This finding has major implications for our understanding of the evolutionary history of *Cardinium* symbionts and suggests that these bacteria may have adapted to living in plant tissues outside the insect host. The genome of the *Cardinium* endosymbiont of the whitefly *Bemisia tabaci* (a phloem feeder like *S. titanus*) showed peculiar traits like genes associated with gliding motility, which may be responsible for the colonization of new hosts^46^, possibly including plants. Further clues to the adaptation to life in the plant tissues should be sought in the available genomes of *Cardinium* and should suggest the possibility of a dual lifestyle of this ubiquitous symbiont.

## Methods

### Insect rearing

Third- and fourth-instar nymphs of *S. titanus* and *E. vitis* were collected during the early summer from vineyards in the Piedmont region of northwestern Italy (Fig. S1) between 2007 and 2012 and reared on healthy grapevine plants in laboratory cages at the Dipartimento di Scienze Agrarie, Forestali e Alimentari (DISAFA) in growth chambers at 25 °C and a photoperiod of 16:8 (L:D) h until adult emergence. *Macrosteles quadripunctulatus* individuals were obtained from laboratory lines reared on oats (*Avena sativa*) in growth chambers with the same temperature and photoperiod conditions.

All experiments were conducted in accordance with the legislation and guidelines of the European Union for the protection of animals used for scientific purposes (http://ec.europa.eu/environment/chemicals/lab_animals/legislation_en.htm). All experimental protocols using animals were approved by the *ad-hoc* Committee of DISAFA of the University of Turin. In addition, all necessary permits were in hand when the research was conducted.

### *Cardinium* inoculation experiments through artificial and plant diets

To observe—under controlled conditions—the injection of bacterial cells by *S. titanus* while feeding, artificial feeding systems were set up (Fig. S2A). A total of 100 newly emerged adults were maintained on the artificial diet for three days according to^16^ (see also SI material and methods). Half of the specimens (50 insects) were reared on the artificial diet supplemented with 300 μg ml^−1^ rifampicin, known to be active against *Cardinium*^9^,^16^.

To confirm bacterial release under semi-natural conditions, a cage system on the grapevine leaves was set up^16^,^47^ with small plastic insect chambers placed on the surfaces of the leaves (Fig. S2B) to force a total of 90 *S. titanus* to feed on small areas of the plants for three days (see also SI material and methods). Half of the leaves were supplied with 300 μg ml^−1^ rifampicin together with a nutritive solution, and the leafhoppers were exposed to these treated leaves for three days.

A total of 35 leaves from grapevine seedlings never exposed to *S. titanus* were used as the control in the molecular analyses. Furthermore, to check for grapevine infection by *Cardinium* in the field, 45 leaves (35 for qPCR and 10 leaf midribs for FISH) from different Barbera plants in Piedmont vineyards with high *S. titanus* incidence were also tested.

### *Cardinium* transmission tests

Two cicadellid species, the phytoplasma vector *M. quadripunctulatus* and the grapevine feeder *E. vitis*, were used to evaluate the actual transfer of the symbiont to recipient insects after the inoculation by *S. titanus* during feeding events (see also SI material and methods). Twenty-five *M. quadripunctulatus* and 25 *E. vitis* were firstly checked by PCR and qPCR (with Card192F/1069R and EndoF1/R3 primer pairs, respectively, as described in SI material and methods) for the actual absence of *Cardinium* in the used populations to verify whether these species were suitable for transmission experiments.

The horizontal transfer of *Cardinium* to *M. quadripunctulatus* was assessed *via* feeding experiments in artificial media chambers (Fig. S2C). Adult donor individuals of *S. titanus* were maintained for three days in groups of 15 insects on sugar solutions in suitable feeding chambers to allow the symbiont to be released in the supplied substrate. They were then replaced by groups of 15 *M. quadripunctulatus* adults maintained for different acquisition periods: one day, three days, and seven days.

The transmission of *Cardinium* to *E. vitis* occurred *via* the grapevine leaves. Single donor *S. titanus* specimens were forced to feed for three days on portions of Barbera grapevine leaves for the release of bacterial cells into the plant tissues and then removed and preserved for subsequent analyses. The same leaf portions were then exposed to *E. vitis* individuals to allow bacterium acquisition for one day, three days, and seven days.

### DNA extraction and PCR-based analyses

Subsequent to the inoculation experiments, total DNA was extracted from the leafhoppers and the respective sugar diets and the leaf portions for molecular analyses. Nucleic acid extraction from the insects and artificial diet was performed as previously reported^47^,^48^, whereas plant DNA was extracted from the leaf portions previously ground with liquid nitrogen in a sterile mortar, according to DNeasy Plant Mini Kit protocol (Qiagen, Italy) instructions.

Quantitative real-time PCR was performed to measure the presence and concentration of *Cardinium* cells in the insects, artificial diet and leaves. In all samples, reactions targeting the 16S rRNA gene of *Cardinium* were carried out. In addition, insect DNA was submitted to qPCR targeting the insect’s 18S rRNA gene to normalize the absolute *Cardinium* density. Furthermore, insect and artificial diet DNA (but not the grapevine DNA due to a non-specific reaction with the plastids of bacterial universal primers) was used in reactions with universal bacterial primers to define the overall bacterial concentration in each sample. The CBR was then calculated to estimate the relative abundances of *Cardinium* in the bacterial community associated with the leafhoppers or with colonizing the diet. qPCR conditions are reported in the SI material and methods.

Subsequent to qPCR screening, 10 *Cardinium*-positive *M. quadripunctulatus* and *E. vitis* individuals were used for PCR screening (SI materials and methods). The obtained PCR products were purified and sequenced (Genechron, Rome, Italy), and the resulting sequences were compared with those in the National Center for Biotechnology Information (NCBI) sequence database by using BLAST (http://www.ncbi.nlm.nih.gov/blast). A phylogenetic analysis was performed using the MEGA 6 software by the neighbor joining method.

### Transmission electron microscopy and *in situ* hybridization

Transmission electron microscopy was carried out on *S. titanus* individuals after dissection in saline solution, as previously described^49^. ISH and FISH analyses were performed on the salivary glands of *S. titanus*, whereas the artificial feeding media, grapevine leaves, and *Cardinum*-recipient *M. quadripunctulatus* and *E. vitis* individuals were analyzed by FISH only.

ISH experiments were carried out by using specific oligonucleotide probes, Card172 and Card1069^50^, 5′ labelled with digoxigenin (DIG). FISH experiments performed on insect tissues and feeding media were carried out with *Cardinium*-specific probes along with a universal bacterial probe, which was employed as a positive control for the hybridization experiment. Conversely, for experiments on plant tissues only, *Cardinium*-specific probes were employed, due to non-specific hybridization of the eubacterial probe with the plastids. For the complete procedures, see also SI materials and methods.

## Additional Information

**How to cite this article**: Gonella, E. *et al.* Plant-mediated interspecific horizontal transmission of an intracellular symbiont in insects. *Sci. Rep.* **5**, 15811; doi: 10.1038/srep15811 (2015).

## Supplementary Material

Supplementary Information

Supplementary Figure S1

Supplementary Figure S2

Supplementary Figure S3

Supplementary Figure S4

Supplementary Figure S5

## Figures and Tables

**Table 1:** *Cardinium* infection in *S. titanus* fed on antibiotic-treated or untreated artificial media or grapevine leaves.

Samples	Avg *Cardinium* infection rate and (SE) (%)*	Avg. *Cardinium* cell number and (SE) *	Range of *Cardinium*cell number	CBR Avg and (SE)*	CBR Range
Without antibiotic					
Insect (on diet)	94.3 (5.7) a	6.41 × 10^6^ (5.83 × 10^6^) ab	<1.66 × 10^1^–1.92 × 10^8^	0.0638 (0.0243)	0.0–0.7253
Diet	62.9 (13.4) abc	3.20 × 10^6^ (2.26 × 10^6^) ab	<1.66 × 10^1^–5.33 × 10^7^	0.0402 (0.0237)	0.0–0.4709
Insect (on leaves)	97.1 (2.9) a	7.74 × 10^6^ (3.30 × 10^6^) a	<1.66 × 10^1^–4.68 × 10^7^	0.1115 (0.0515)	0.0–0.7257
Grapevine	68.6 (7.4) abc	8.70 × 10^3^ (4.37 × 10^3^) bc	<1.66 × 10^1^–8.06 × 10^4^	ND*	ND*
With antibiotic					
Insect (on diet)	94.3 (5.7) a	4.94 × 10^5^ (2.04 × 10^5^) ab	<1.66 × 10^1^–3.35 × 10^6^	0.0463 (0.0247)	0.0–0.4895
Diet	22.9 (6.8) d	3.54 × 10^2^ (2.87 × 10^2^) c	<1.66 × 10^1^–1.50 × 10^3^	0.0018 (0.0014)	0.0–0.0059
Insect (on leaves)	80.7 (10.9) ab	2.06 × 10^6^ (1.01 × 10^6^) ab	<1.66 × 10^1^–1.92 × 10^7^	0.0383 (0.0211)	0.0–0.147
Grapevine	45.7 (12.1) bcd	2.23 × 10^3^ (1.53 × 10^3^) bc	<1.66 × 10^1^–9.82 × 10^3^	ND*	ND*
Grapevine controls					
Seedlings	Nd*	<1.66 × 10^1^ d	Nd*	Nd*	Nd*
Healthy plants	Nd*	<1.66 × 10^1^ d	Nd*	Nd*	Nd*
Field plants	34.3 (9.5) cd	3.16 × 10^2^ (2.61 × 10^2^) c	<1.66 × 10^1^–5.83 × 10^3^	ND*	ND*

The average *Cardinium* titre is expressed as *Cardinium* cells [=*Cardinium* 16S rRNA gene copies^51^]/sample]. Samples are defined as follows: single insect, single diet unit (300 μl sugar solution), 100 mg of grapevine leaf. Values below the detection limit (1.66 × 10^1^ *Cardinium* cells/sample) were considered negative. For grapevine leaves, three controls are indicated: seedlings are vines employed for inoculation trials before use; healthy plants are grapevines never exposed to *S. titanus*; field plants are grapevine plants collected in vineyards with high *S. titanus* pressure. ANOVA tests were carried out comparing *Cardinium* infection rates and concentrations separately. Within the same column letters indicate significantly different values (p < 0.05). N = 35 for all groups. *Avg: Average values; SE: Standard Error; Nd: Not Detectable (*Cardinium* below detection limit 1.66 × 10^1^); ND: Not determined (qPCR with bacterial universal primer not determined due to the masking effect of amplification of plastid 16S rRNA genes).

**Table 2:** Results of *Cardinium* transmission experiments to *M. quadripunctulatus* and *E.vitis*.

	Duration of *Cardinium* acquisition in the recipient	Donor no. samples	Donor avg *Cardinium* titre and (SE)*	Recipient no. samples	Recipient avg *Cardinium* infection rate (%) and SE (%)*	Recipient avg *Cardinium* titre and (SE)*	*Cardinium* titre range	Recipient CBR avg (%) and SE (%)*
ST to MQ	control	—	—	25	Nd	< 1.66 × 101 b	Nd	Nd
	1 day	25	2.30 × 10^8^ (7.72 × 10^7^)	30	53.33 (9.19) a	9.21 × 10^1^ (7.30 × 10^-1^) a	<1.66 × 10^1^–2.26 × 10^2^	0.0002 (0.00004)
	3 days	28	3.18 × 10^8^ (1.03 × 10^8^)	30	53.33 (6.91) a	3.34 × 10^2^ (8.07 × 10^1^) a	<1.66 × 10^1^–2.20 × 10^3^	0.0011 (0.0002)
	7 days	27	6.94 × 10^6^ (5.35 × 10^5^)	29	75.56 (1.43) b	8.17 × 10^1^ (7.56 × 10^0^) a	<1.66 × 10^1^–5.78 × 10^2^	0.0001 (0.00002)
ST to EV	control	—	—	25	Nd	<1.66 × 10^1^ d	Nd	Nd
	1 day	30	1.55 × 10^6^ (4.14 × 10^5^)	30	73.33 (5.58) ab	1.23 × 10^5^ (1.77 × 10^4^) ab	<1.66 × 10^1^–1.15 × 10^6^	0.0367 (0.0067)
	3 days	25	1.30 × 10^5^ (3.64 × 10^4^)	25	27.38 (5.73) bc	1.89 × 10^2^ (2.66 × 10^1^) c	<1.66 × 10^1^–3.83 × 10^2^	0.0151 (0.0028)
	7 days	27	2.91 × 10^6^ (9.47 × 10^5^)	27	66.67 (2.14) ab	3.53 × 10^3^ (5.20 × 10^2^) bc	<1.66 × 10^1^–2.32 × 10^4^	0.0436 (0.0080)

The average *Cardinium* titre determined by qPCR is expressed as *Cardinium* cells [=*Cardinium* 16S rRNA gene copies^51^] per insect. Values below the detection limit (1.66 × 10^1^) were considered negative. The infection rate is expressed as percentage *Cardinium*-positive individuals related to the total tested specimens. Donor = *S. titanus* (ST); recipient = *M. quadripunctulatus* (MQ) or *E. vitis* (EV). The controls are *M. quadripunctulatus* or *E.vitis* individuals never submitted to co-feeding experiments. ANOVA tests were carried out comparing *Cardinium* infection rates and concentrations for *M. quadripunctulatus* and *E. vitis* separately. For each experiment, different letters indicate significantly different values (p < 0.05). *Avg: average values; SE: Standard Error; Nd: Not Detectable (*Cardinium* below detection limit 1.66 × 10^1^).
