# Supplementary material for: Plant-mediated interspecific horizontal transmission of an intracellular symbiont in insects
Source: Sci Rep. 2015 Nov 13;5:15811. doi: 10.1038/srep15811 (PMC4643326; doi:10.1038/srep15811)
Supplement: Supplementary Information [file srep15811-s1.pdf]

SUPPLEMENTARY INFORMATION

Plant-mediated interspecific horizontal transmission of an intracellular symbiont in insects

Elena Gonella[1], Massimo Pajoro[1a], Massimo Marzorati[2b], Elena Crotti[2], Mauro Mandrioli[3], Marianna Pontini[1], Daniela Bulgari[4], Ilaria Negri[1], Luciano Sacchi[5], Bessem Chouaia[2], Daniele Daffonchio[2,6]*, and Alberto Alma[1]*

[1]Dipartimento di Scienze Agrarie, Forestali e Alimentari (DISAFA), Università degli Studi di Torino, 10095 Grugliasco, [2]Dipartimento di Scienze per gli Alimenti, la Nutrizione, l'Ambiente (DeFENS), Università degli Studi di Milano, 20133 Milan, [3]Dipartimento di Scienze della Vita, Università di Modena e Reggio Emilia, 41100 Modena, [4]Dipartimento di Scienze Agrarie e Ambientali - Produzione, Territorio, Agroenergia, Università degli Studi di Milano, 20133 Milan, [5]Dipartimento di Biologia e Biotecnologie "L. Spallanzani", Università degli Studi di Pavia, 27100 Pavia, [6]King Abdullah University of Science and Technology, BESE Division, Thuwal, 23955-6900, Kingdom of Saudi Arabia.

[a]Present address: Dipartimento di Scienze Veterinarie e Sanità Pubblica (DIVET), Università degli Studi di Milano, 20133 Milan, Italy

[b]Present address: Laboratory for Microbial Ecology and Technology (LabMET), Ghent University, B9000 Ghent, Belgium

*To whom correspondence should be addressed. E-mails: alberto.alma@unito.it; daniele.daffonchio@kaust.edu.sa

SUPPLEMENTARY INFORMATION

SUPPLEMENTARY METHODS

Set up of feeding systems for *Cardinium* inoculation and transmission trials

For detection of *Cardinium* cells in artificial diet, a liquid feeding medium was prepared with 0.5% sucrose (w/v) dissolved in 1X TE buffer (pH8) and sterilized by filtering (0.2-µm-pore-size filter). When required, 300µg ml^{-1} rifampicin were added to the solution.

In inoculation experiments 2 ml Eppendorf tubes cut at the base were employed as feeding cages for single *S. titanus* individuals. The tube caps were filled with 300 µl of sterile sugar solution and sealed with parafilm. After placing the insects in the tube, cotton was inserted at the base to guarantee suitable air flux and moisture conditions (Fig. S2A). Insects were maintained singularly for three days in such cages placed horizontally in growth chambers at 25°C and a photoperiod of 16:8 (L:D) h. An attractive yellow film was put in front of the tube caps. At the end of the experiments, all the *S. titanus* individuals and the feeding media were collected. For each treatment 35 insects and the respective feeding media were preserved at -20°C for quantitative PCR (q-PCR) analyses, while 10 and 5 individuals were respectively used for *in situ* hybridization (following salivary gland dissection) along with aliquots of their feeding media, and transmission electron microscopy (TEM).

In transmission experiments to *M. quadripunctulatus* 50 ml tubes were used as feeding chambers for groups of 15 individuals. Eight millilitres of sugar solution were used to fill the tube cap before sealing with parafilm. The base of tubes was cut and replaced by cotton (Fig. S2C). Donor insects were reared in these cages for three days as described above, and successively collected and used for further analyses; groups of 15 *M. quadripunctulatus* adults were fed with the same feeding media for one to seven days. Three replicates were carried out per each acquisition time. At the end of the experiments, the insects were collected for analysis: 15 individuals were used for FISH and the remaining for qPCR screening.

To detect *Cardinium* cells injected by *S. titanus* in grapevine leaves, ten vine seedlings (cultivar Barbera) were firstly checked by molecular screening for the absence of *Cardinium* and then used. The absence of *Cardinium* was verified by qPCR with the MoCardRT-F2/R1 primer pair (5, see below) after DNA extraction of two randomly-sampled leaves with DNeasy Plant Mini Kit protocol (Qiagen, Italy) according to manufacturer's instructions. Single leaves were collected and isolated by placing petioles inside Eppendorf tubes containing the following nutritive solution: 0.3875 g l^{-1} NaH_2PO_4, 2.59 g l^{-1} KNO_3, 5.45 g l^{-1} $Ca(NO3)_2$, 3.65 g l^{-1} $MgSO_4$, 0.52 g l^{-1} Na_2SO_4, (9) to maintain leaf turgor, and sealed with parafilm. When required, 300µg ml^{-1} rifampicin were added to the solution. The feeding cages were made with small plastic insect chambers placed on the surface of leaves (Fig. S2B). *S. titanus* specimens were maintained for three days in these cages in the same conditions described above for both inoculation trials and transmission experiments to *E. vitis* and then removed. At the end of the inoculation experiment, 35 insects and the respective leaf portions for each treatment were collected and preserved at -20°C for qPCR analyses, whereas 10 individuals and their leaves were used for *in situ* hybridization. For transmission trials to *E. vitis*, recipient leafhoppers were reared in the same cages previously exposed to donor *S. titanus* for 1 day, 3 days, and 7 days; subsequently they were collected and preserved at -20°C. Groups of 40-45 recipient individuals were taken for each treatment: 15 insects were used for FISH and the remaining for qPCR.

Quantitative real-time and conventional PCR

Quantitative real-time PCR was performed on a Chromo4 real-time detector (Bio-Rad, Milan, Italy) with IQTM SYBR® Green Supermix (Bio-Rad). The reactions were performed with the EndoF1/R3 primer pair, targeting the 16SrRNA gene of *Cardinium* (8). The reaction conditions were: an initial denaturation at 95°C for 3 min was followed by 50 cycles consisting of denaturation at 95°C for 30 sec, annealing at 60°C for 30 sec, and extension at 72°C for 1 min. The average *Cardinium* 16S rRNA gene copy numbers / sample were considered the same as the average

Cardinium cells because this gene is estimated to be in single copy in the *Cardinium* genome (10, 12). In addition to normalize the absolute *Cardinium* density in insects a qPCR targeting the insect's 18S rRNA gene (MqFw / MqRv) was used (7), consisiting of an initial denaturation at 95°C for 3 min, followed by 50 cycles consisting of denaturation at 95°C for 45 sec, annealing at 65°C for 1 min, and extension at 72°C for 1 min. Normalized *Cardinium* cell numbers were calculated per pg of insect 18Sr RNA gene, whereas for the diets the normalized *Cardinium* concentration was calculated per whole sample (expressed as ng of total DNA obtained from 300 μl of sugar solution). Moreover, as the use of EndoF1/R3 primer set resulted in non-specific reaction with the plastids, quantitative real-time PCR for plant samples was only carried out with the specific primers MoCardRT-F2/R1 (5) as follows: after an initial denaturation at 95°C for 3 min, 50 cycles consisting of denaturation at 95°C for 30 sec, annealing at 65°C for 30 sec, and extension at 72°C for 1 min were performed. Results from plant samples were expressed as cells of *Cardinium* per 100 mg of leaf. To assess the comparability of values obtained with different primer sets, 12 samples of insects and feeding media (3 *S. titanus*, 3 *M. quadripunctulatus*, 3 *E. vitis*, 3 artificial diets) were tested with EndoF1/R3 and then with MoCardRT-F2/R1, and the results where compared. The detection limit was 1.66×10^1 copies of *Cardinium* 16S rRNA gene per sample (11).

For insects and feeding media, reactions with bacterial universal primers 357F and 907R were performed as well, as previously described (13), to define the overall bacterial concentration in each sample. Reactions were performed with the following conditions: 95°C for 3 min; 50 cycles of 95°C for 30 sec, 55°C for 50 sec, and 72°C for 1 min.

Subsequent to qPCR screening, 10 *Cardinium*-positive *M. quadripunctulatus* and *E. vitis* individuals were used for PCR with the primers Card192F (5'-TTAGCGCATGCTAGAAAGAT-3') and Card1069R (5'- CACCTTGTATTCCGTCC-3'), targeting the 16SrRNA gene of the symbiont. PCR conditions were the following: 94°C for 4 min; five cycles of 94°C for 1 min, 50°C for 45 sec, and 72°C for 1 min; 30 cycles of 94°C for 45 sec, 55°C for 45 sec, and 72°C for 1 min; and 7 min at 72°C.

Statistical analysis

To compare data obtained in this work, the percentages of infected individuals detected in release and transmission experiments for every tested species and acquisition period were submitted to arcsine square root transformation; qPCR data relative to *Cardinium* cells concentration observed within each experiment were log-transformed after adding the constant 10. All transformed data were analyzed with replicates of five samples by one-way analysis of variance (ANOVA). In addition, means were separated by Tukey test ($P<0.05$) when variance homogeneity was satisfied (Levene test, $P<0.05$).

***In situ* hybridization and histological stain**

For ISH experiments insect tissues were fixed in 4% phosphate-buffered paraformaldehyde overnight at 4°C, dehydrated, cleared by using an EtOH-xylene series, and embedded in paraffin. Five-micrometers thick sections were mounted on poly (L-lysine)-coated microscope slides. After dewaxation and rehydration in a xylene-EtOH series, the sections were subjected to the following steps: pre-treatment with proteinase K (10 µg ml^{-1}), refixation in 4% paraformaldehyde, treatment with 0.2 M HCl, dehydration through an EtOH series, and air drying. Hybridization was performed for 4h at 42°C in a dark moisture chamber in a buffer (0.9 M NaCl, 20 mM Tris-HCl, 30% formamide) containing the two specific DIG-labeled probes (each at a concentration of 2 ng μl^{-1}). Slides were washed in a second buffer (20 mM Tris-HCl, 0,1 M NaCl, 0.01% sodium dodecyl sulfate) at 44°C for 10 min. Maximally stringent hybridization and washing conditions were chosen using optimization procedures previously described (4). Specific binding of probes was detected incubating slides with anti-DIG antibodies conjugated with horseradish peroxidase [Polyclonal Rabbit anti-DIG/HRP, Rabbit F (ab')] by using the manufacturer's instructions (Dako, Milan, Italy). Staining was performed with 3-amino-9-ehtylcarbazole (AEC) chromogenic substrate (Dako, Milan, Italy). Tissue sections were then permanently mounted on aqueous mounting medium (Bio-Optica,

Milan, Italy) and observed under light microscope. All experimental procedures were conducted in RNAse-free conditions. A positive control experiment with the universal bacterial probe EUB338 (3) was also performed applying the same conditions. In order to better characterise the insect's histology, haematoxylin/eosin stain and Periodic acid–Schiff (P.A.S.) stain were also applied to some tissue sections.

FISH experiments performed on insect tissues and feeding media were carried out with the *Cardinium*-specific probes labelled with Cy5 (indodicarbocyanine) (absorption and emission at 650 nm and 670 nm, respectively) or Texas Red (absorption and emission at 595 nm and 620 nm, respectively) along with a universal bacterial probe EUB388, labelled with fluorescein isothiocyanate (FITC, absorption/ emission at 494/520 nm) or Texas Red, which was employed as a positive control for the hybridization experiment. Conversely, for experiments on plant tissues only *Cardinium*-specific probes were employed, due to a non-specific hybridization of the EUB338 probe with the plastids.

Whole mount FISH experiments were performed on salivary glands dissected in a sterile saline solution, then fixed for 2 min at 4°C in 4% paraformaldehyde and washed in PBS. All hybridization experiment steps were performed as previously described (2). After hybridization, the samples were mounted in anti-fading medium and then observed in a laser scanning confocal microscope SP2-AOBS (Leica).

To perform FISH experiments on the artificial feeding media provided to *S. titanus*, the sugar solutions were filtered on polycarbonate membrane and fixed with ethanol and 4% paraformaldehyde according to (6). After fixation, the filter sections were hybridized with the same probes used for insect samples. Hybridization was carried out in dark conditions for 2 hours at 46°C, in the following hybridization buffer: 0.9 M NaCl, 20 mM TrisHcl, 0.01% SDS, 35% formamide; 10 ng of each probe were added. Afterwards, filter sections were washed in washing buffer (0.9M NaCl, 20mM TrisHcl, 0.01% SDS, 35% formamide) at 50°C and then in MilliQ water at 4°C. Samples were mounted in anti-fading medium, and subsequently observed using a Leica DM4000B

equipped with Leica filter set G/R (excitation filter, BP 490/20 nm; dichromatic mirror, 505; emission filter, LP 525/20 and excitation filter, BP 575/30 nm; dichromatic mirror, 600; emission filter, LP 635/40).

For FISH experiments on grapevine leaf tissues, midribs portions were fixed in 4% paraformaldehyde, cut in 50-70 µm slides with a vibroslice (World Precision Instruments, Germany) without the paraffin-embedding step. The hybridization was carried out as previously reported (1), with the *Cardinium*-specific Cy5-labelled probe, with the following modification: an overnight hybridization was carried out at 37°C. After hybridization, the samples were mounted in antifading medium and then visualized by video-confocal microscopy (Nikon, Italy).

All the hybridization experiments included negative controls involving either treatment with RNAse prior to probe hybridization step or in the absence of probes.

References

1. Bulgari, D., Casati, P. & Faoro, F. *et al*. Fluorescence in situ hybridization for phytoplasma and endophytic bacteria localization in plant tissues. *J. Microbiol. Meth.* **87,** 220-223 (2011).

2. Crotti, E. *et al*. *Asaia*, a versatile acetic acid bacterial symbiont, capable of cross-colonizing insects of phylogenetically distant genera and orders. *Environ. Microbiol.* **11,** 3252-3264 (2009).

3. Fuchs, B. M. *et al*. Flow cytometric analysis of the *in situ* accessibility of *Escherichia coli* 16S rRNA for fluorescently labeled oligonucleotide probes. *Appl. Environ. Microbiol.* **12,** 4973-4982 (1998).

4. Hugenholtz, P. *et al*. in: Lieberman, B.A. (Ed.) Methods in Molecular Biology, **176,** 29-41 (Humana Press Inc.) (2001)

5. Jeyaprakash, A. & Hoi, M. A. Real-time PCR reveals endosymbiont titer fluctuations in *Metaseiulus occidentalis* (Acari: Phytoseiidae) colonies held at different temperatures. *Florida Entomol.* **93,** 464-466 (2010).

6. Le Gullec, D. Ultrastructural *in situ* hybridization: a review of technical aspects. *Biol. Cell* **90,** 297-306 (1998).

7. Marzachì, C. & Bosco, D. Relative quantification of chrysanthemum yellows (16Sr I) phytoplasma in its plant and insect host using real-time polymerase chain reaction. *Mol. Biotechnol.* **30,** 117-127 (2005).

8. Marzorati, M. *et al.* A novel *Bacteroidetes* symbiont is localized in *Scaphoideus titanus*, the insect vector of Flavescence Dorée in *Vitis vinifera*. *Appl. Environ. Microbiol.* **72,** 1467-1475 (2006).

9. Moutous, G. & Fos, A. Essais de rhizogénèse chez la feuille de vigne isolée. *Rev. Zool. Agric. Pathol. Vég.* **1,** 27-28 (1973).

10. Nakamura, Y. *et al.* Differentially expressed genes in silkworm cell cultures in response to infection by *Wolbachia* and *Cardinium* endosymbionts. *Insect Mol. Biol.* **20,** 279-289 (2011).

11. Pajoro, M. *et al.* Investigation on the life cycle of ST1-C, the endosymbiont *of Scaphoideus titanus*. *Bull. Insectol.* **61,** 217-218 (2008).

12. Penz, T. *et al.* Comparative genomics suggests an independent origin of cytoplasmic incompatibility in *Cardinium hertigii*. *PLoS Genet.* **8,** e1003012 (2012).

13. Raddadi, N. *et al.* '*Candidatus* Liberibacter europaeus' sp. nov. that is associated with and transmitted by the psyllid *Cacopsylla pyri* apparently behaves as an endophyte rather than a pathogen. *Environ. Microbiol.* **13,** 414-426 (2011).

SUPPLEMENTARY INFORMATION

SUPPLEMENTARY FIGURE LEGENDS

Fig. S1 Location of the sampling sites for *S. titanus* and *E. vitis* collection in the Piedmont region (black asterisks). The inset indicates the location of the region in Italy (black dot). The sampling sites were situated in the Asti Province, municipality of Portacomaro (1) (44°58'12.6"N 8°15'07.3"E), and in the Torino Province, municipalities of Prascorsano (2) (45°21'18.2"N 7°37'02.1"E), Grugliasco (3) (45°04'01.3"N 7°35'32.4"E), and Bricherasio (4) (44°49'52.8"N 7°18'06.2"E). The figure was created by modifying the maps of Piedmont and Italy, respectively downloaded from http://d-maps.com/carte.php?num_car=8251&lang=it and http://d-maps.com/carte.php?num_car=14531&lang=it.

Fig. S2 Feeding systems for *Cardinium* inoculation and transmission trials used in this work. A) Feeding cages for single *S. titanus* individuals employed to test the release of *Cardinium* in artificial media. B) Feeding cages set up on grapevine leaves to assess *Cardinium* inoculation to the plant by *S. titanus* and the transmission to *E. vitis*. C) Feeding cages for groups of leafhoppers used for transmission trials to *M. quadripunctulatus* via artificial diet.

Fig. S3 FISH negative controls applied to the *S. titanus* salivary glands showed in A) and C) (interferential contrast micrographs). B) Sample treated with RNAse prior to probe hybridization step. D) No probe control. Bars = 75 μm.

Fig. S4 FISH negative controls carried out on *M. quadripunctulatus* and *E. vitis* used in *Cardinium* transmission trials. A-D) Interferential contrast micrographs of intestines of *M. quadripunctulatus* (A, B) and *E. vitis* (C, D). Bars = 75 μm. E-H) DAPI staining of the same gut portions. The organs

were submitted to the following controls: RNAse treatment before hybridization (I, K), hybridization in the absence of probes (J, L).

Fig. S5 Phylogenetic analysis of the *Cardinium* strains recorded in *M. quadripunctulatus* and *E. vitis*. *Cardinium* phylogenetic groups are indicated. The phylogenetic tree was constructed based on the almost complete 16S rRNA gene by the neighbor joining method with the software MEGA 6. Numbers at each node represent percentages of bootstrap replications calculated from 1,000 replicate trees. The scale bar represents the sequence divergence. The Bacteroidetes 'Ca. Sulcia muelleri' (JX514697), symbiont of many Auchenorrhncha, was used as an outgroup.
