# Supplementary figures and images for: Plant-mediated interspecific horizontal transmission of an intracellular symbiont in insects
Source: Sci Rep. 2015 Nov 13;5:15811. doi: 10.1038/srep15811 (PMC4643326; doi:10.1038/srep15811)

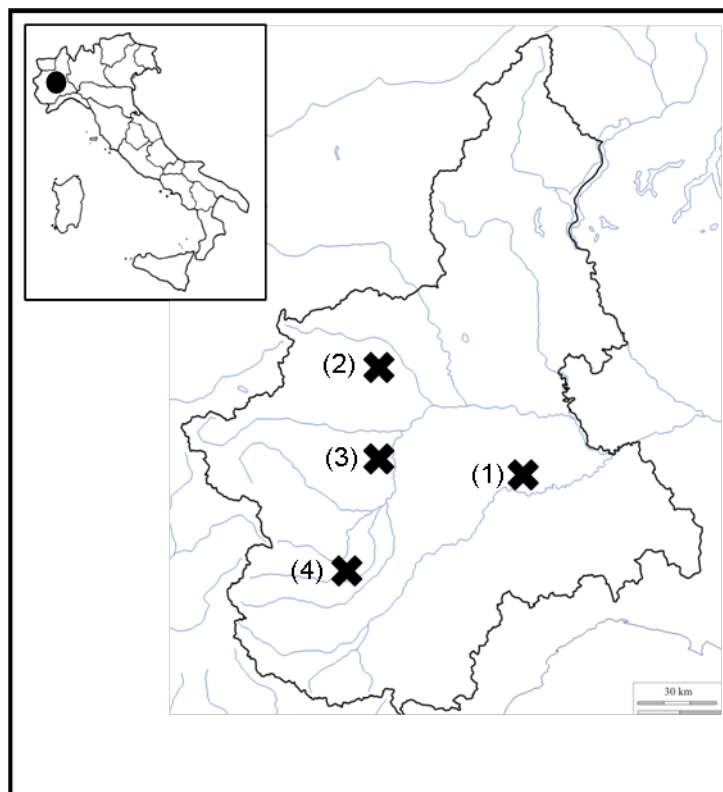

Supplement: Supplementary Figure S1 [file srep15811-s2.pdf]

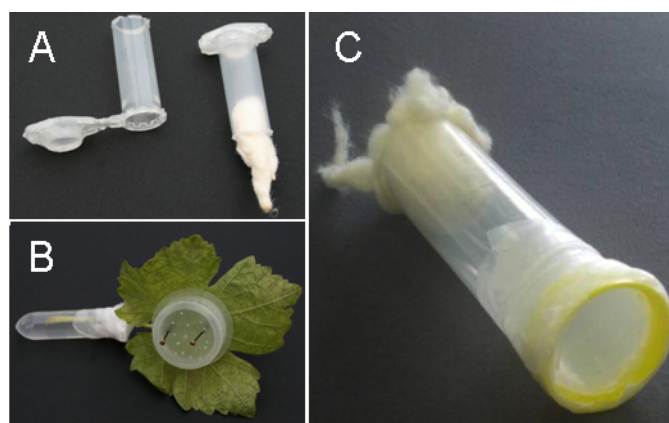

Supplement: Supplementary Figure S2 [file srep15811-s3.pdf]

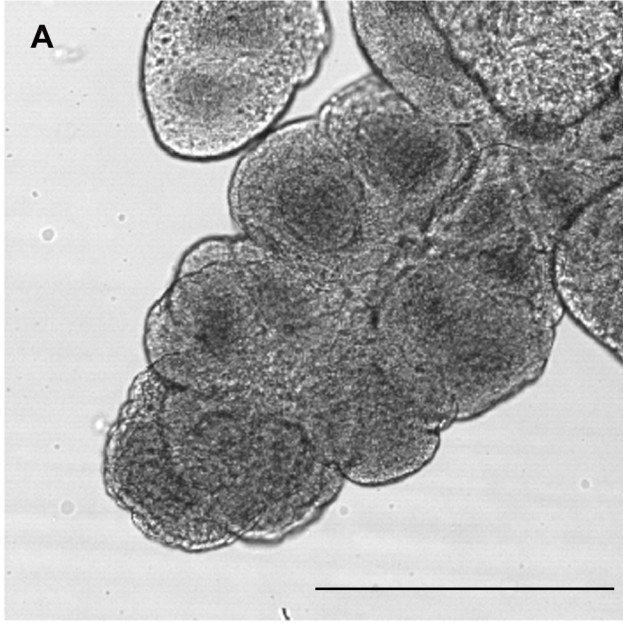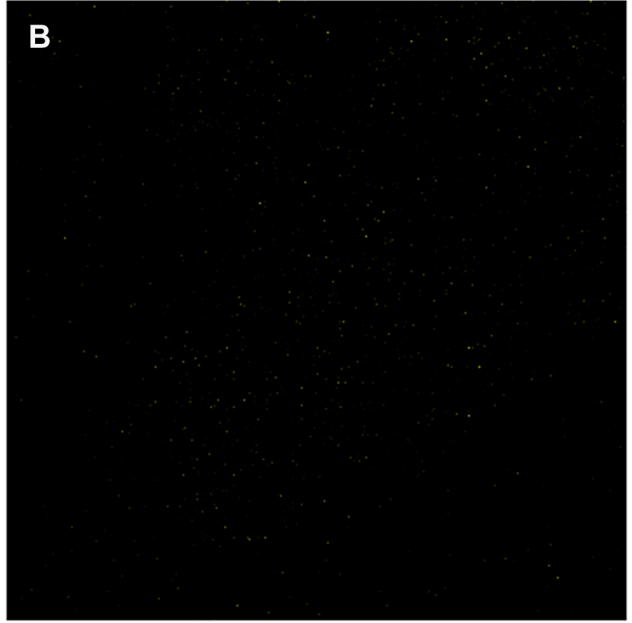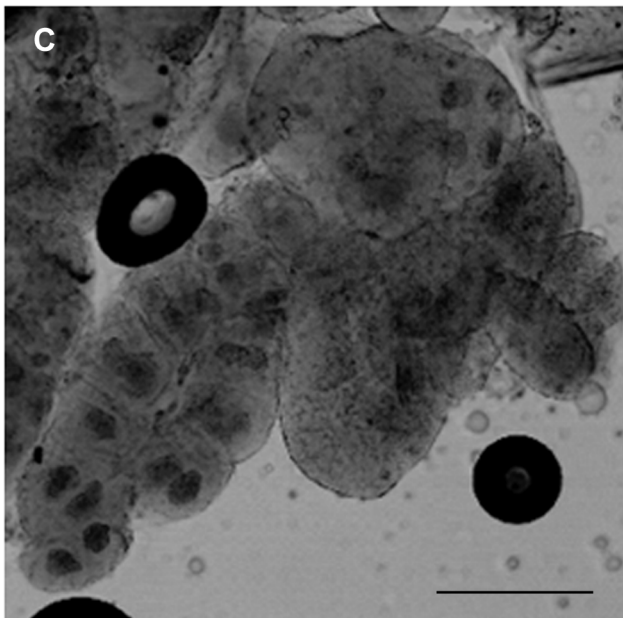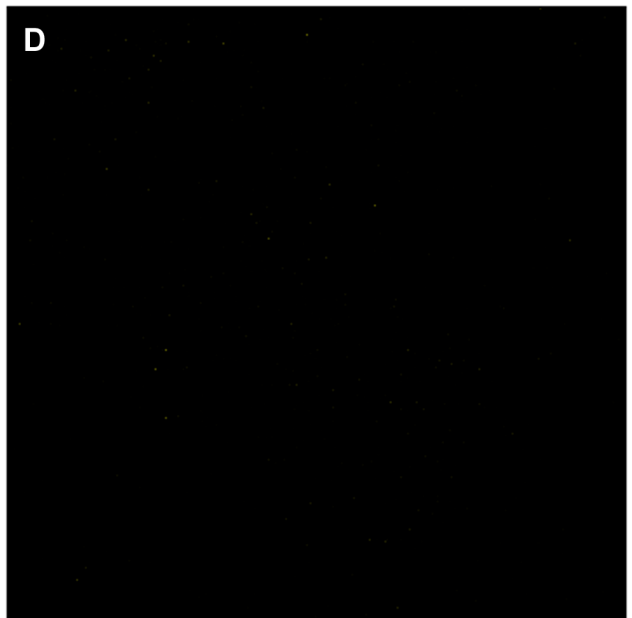

Supplement: Supplementary Figure S3 [file srep15811-s4.pdf]

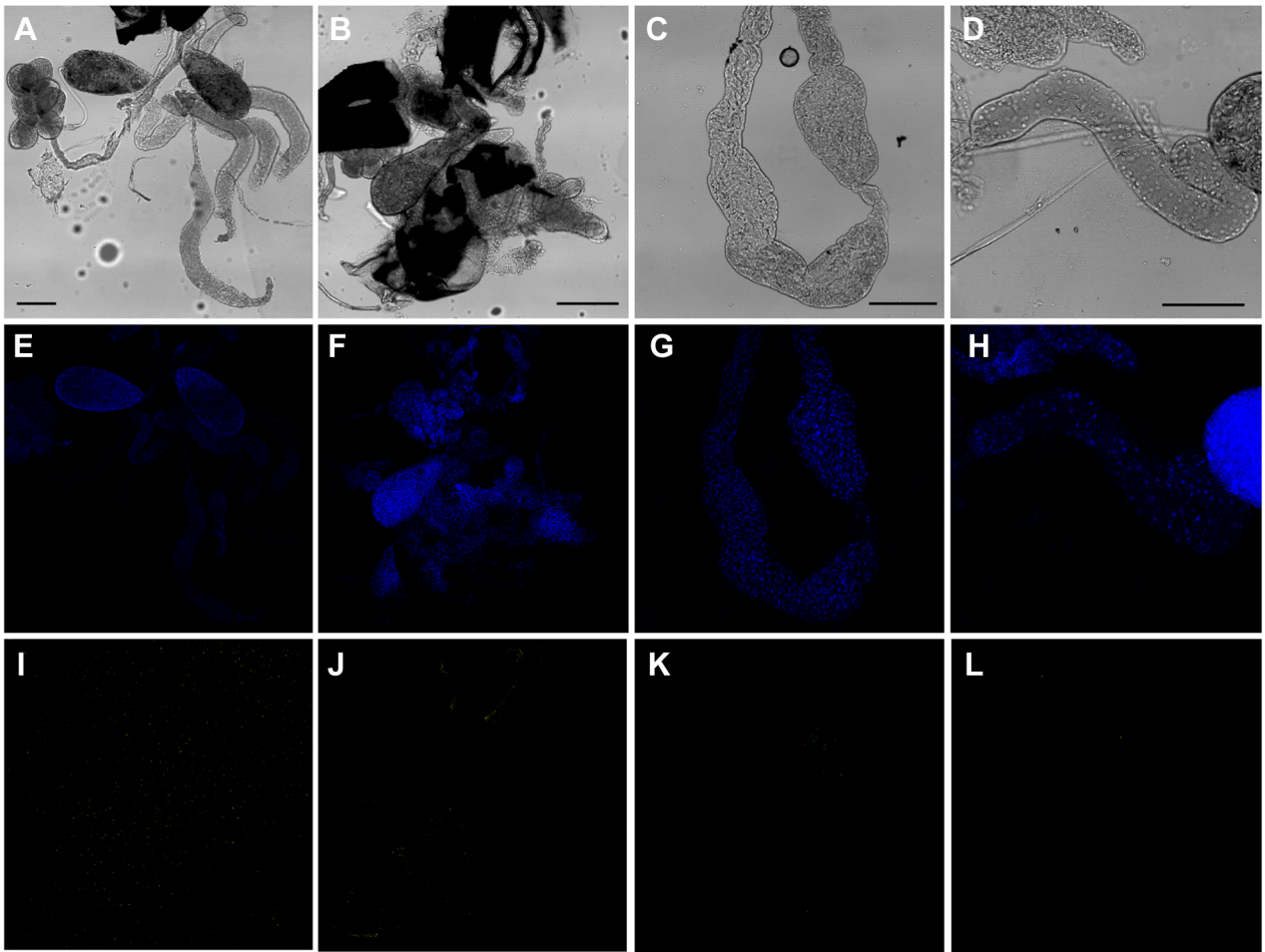

Supplement: Supplementary Figure S4 [file srep15811-s5.pdf]

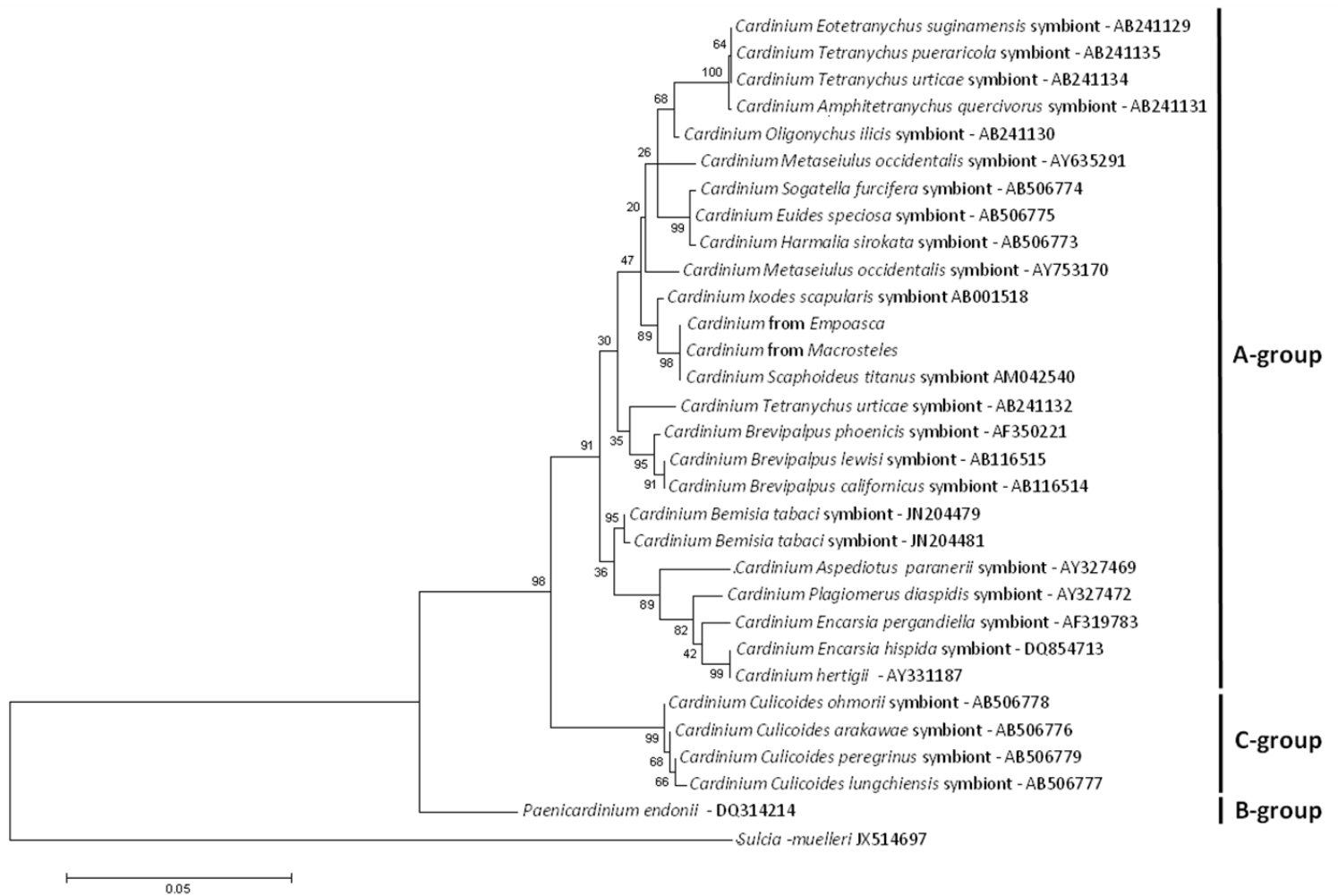

Supplement: Supplementary Figure S5 [file srep15811-s6.pdf]
